# Caregivers’ perception of malaria and treatment-seeking behaviour for under five children in Mandura District, West Ethiopia: a cross-sectional study

**DOI:** 10.1186/s12936-017-1798-8

**Published:** 2017-04-08

**Authors:** Israel Mitiku, Adane Assefa

**Affiliations:** Department of Public Health, College of Medicine and Health Science, Wollo University, P. O. Box: 1145, Dessie, Ethiopia

**Keywords:** Malaria, Caregivers’ perception, Treatment-seeking, Ethiopia

## Abstract

**Background:**

Early diagnosis and prompt malaria treatment is essential to reduce progression of the illness to severe disease and, therefore, decrease mortality particularly among children under 5 years of age. This study assessed perception of malaria and treatment-seeking behaviour for children under five with fever in the last 2 weeks in Mandura District, West Ethiopia.

**Methods:**

A community based cross-sectional study was conducted among 491 caregivers of children under five in Mandura District, West Ethiopia in December 2014. Data were collected using interviewer-administered questionnaires. Data were entered into Epi Info version 7 and analysed using SPSS version 20. Multiple logistic regression analyses were conducted to identify the determinants of caregivers’ treatment-seeking behaviour.

**Results:**

Overall, 94.1% of the respondents perceived that fever is the most common symptom and 70% associated mosquito bite with the occurrence of malaria. Of 197 caregivers with under five children with fever in the last 2 weeks preceding the study 87.8% sought treatment. However, only 38.7% received treatment within 24 h of onset of fever. Determinants of treatment-seeking include place of residence (rural/urban) (AOR 2.80, 95% CI 1.01–7.70), caregivers age (AOR 3.40, 95% CI 1.27–9.10), knowledge of malaria (AOR 4.65, 95% CI 1.38–15.64), perceived susceptibility to malaria (AOR 3.63, 95% CI 1.21–10.88), and perceived barrier to seek treatment (AOR 0.18, 95% CI 0.06–0.52).

**Conclusions:**

Majority of the respondents of this study sought treatment for their under five children. However, a considerable number of caregivers first consulted traditional healers and tried home treatment, thus, sought treatment late. Living in rural village, caregivers’ age, malaria knowledge, perceived susceptibility to malaria and perceived barrier to seek treatment were important factors in seeking health care. There is a need to focus on targeted interventions, promote awareness and prevention, and address misconceptions about childhood febrile illness.

## Background

Malaria remains a major killer of children, particularly in sub-Saharan Africa (SSA), taking the life of a child every 2 min. According to the latest estimates, 214 million cases of malaria occurred globally in 2015 and the disease led to 438,000 deaths [1]. It is one of the major diseases of poor people in developing countries and one of the leading causes of avoidable death, especially in children and pregnant women [2]. In 2015, malaria was the fourth highest cause of death, accounting for 10% of child deaths in SSA [1]. Ethiopia is one of the countries accounting for 90% of the estimated number of *Plasmodium falciparum* infections in SSA in 2013 [3].

Despite the current efforts to control malaria in Ethiopia, it is still one of the main public health problems in the country. It is ranked as the leading communicable disease in the country, accounting for about 30% of the overall Disability Adjusted Life Years lost. Approximately, 68% of the total population of 84.3 million lives in areas at significant risk of malaria. The distribution and transmission of malaria in Ethiopia varies from place to place [4].

When prevention fails, effective malaria case management is key for preventing morbidity and mortality for children under five [3, 5]. The World Health Organization (WHO) recognizes that early diagnosis and prompt treatment, within 24 h of onset of symptoms, is an essential element of malaria control. Evidences show that timely and appropriate treatment has resulted into reduced severe morbidity and mortality among children under the age of 5 years [6]. Despite this, across SSA, only a small proportion of malaria patients, including children, receive prompt and effective treatment [3]. Evidence shows that most malaria related deaths in malaria affected countries occur at home without receiving appropriate medical care, and when care is sought, it is often delayed [7, 8]. According to the 2011 Ethiopian Demographic and Health Survey (EDHS) among children with fever had had fever in the 2 weeks preceding the survey, only one-fourth sought advice or treatment for the fever at a health facility or health provider [9].

The ability of caregivers to recognize and seek appropriate care for the common childhood illnesses like malaria is instrumental in reducing child deaths in low-and middle income countries (LMICs). Early treatment of childhood malaria depends upon mothers’ perception about malaria and prompt recognition of signs and symptoms of the disease and treatment-seeking behaviour [10, 11]. Studies have reported poor practice of health seeking among caregivers of under five children mainly due to caregivers not recognizing signs of childhood illness for seeking care immediately for common childhood illnesses including fever [12]. Failure of individuals to acknowledge that something is wrong or vulnerable to a disease may result into delay in presentation to a health care provider [13].

Health Believe Model (HBM) is one of the most important health behavioural theories [14]. A central concept in the HBM is the ‘perceived susceptibility’, which refers to the perceived chance of acquiring a condition (in this article also referred to as ‘perceived susceptibility’). The ‘perceived susceptibility’ and the ‘perceived severity’ leads to the formation of ‘perceived barrier’ of a certain condition. The likelihood of performing a certain health behaviour is directly linked to the perceived threat, the perceived benefits and barriers of the suggested behaviour change, the self-efficacy and the cues to action [14, 15].

Malaria treatment-seeking behaviour in under five children has been reported to be associated with socio-economic, demographic and personal factors [16–19]. However, there are limited studies pertaining to perceptions about malaria, treatment-seeking for under five children and associated factors at community level in Ethiopia. Understanding the local perceptions of malaria and its influence on health-seeking behaviour from the community’s point of view is critical and relevant to increase community awareness of the problem as well as the importance of early diagnosis and prompt treatment of malaria. The findings of this study assist programme planners and health educators to target and tailor malaria prevention programmes.

## Methods

### Study design and study area

A community based cross-sectional survey was conducted in December 2014 in Mandura District in Western Ethiopia. Mandura is one of the 20 districts in the Benishangul-Gumuz Region of Ethiopia. The region is one of the two regions of the country where the risk of malaria is highest in almost the entire region [4]. The district is situated at the distance of 530 km from Addis Ababa (the capital city of the country). It is administratively organized into 20 kebeles (the lowest administrative units in the country).

According to the 2007 Ethiopian national census, the population of Mandura District was 40,746, of which 8122 were under five children. The majority of the inhabitants practiced traditional beliefs, with 47.76% of the population reporting they observed this belief, while 39.26% of the population said they practiced Ethiopian Orthodox Christianity, and 7.59% were Muslim [20]. The district lies at an altitude between 1050 and 1400 m above sea level, and has an average temperature 31–37 °C and annual rainfall of 900–1200 mm.

### Sample size and sampling procedure

The required sample size was calculated using a single population proportion formula considering the following assumptions: a 95% confidence level, 5% margin of error, an estimated proportion that 80.87% of caregivers are aware of the cause of malaria [21], considering 5% non-response rate and two design effect. The resulting estimated sample size for the study was 503 households.

A two-stage sampling technique was used. First, six kebeles were selected by using simple random sampling from 20 kebeles in the district. Then, the total sample size was allocated proportionally to each of the kebeles based on the number of households with children under the age of five. A sampling frame for list of households with children under the age of five from each selected kebele were prepared in consultation with health extension workers. Then the total sample size was allocated proportional to the size of the households with children under the age of five at each kebele. In each selected kebele, a sample of households with children under the age of five was selected through simple random sampling using lottery methods. The desired target population for the study were caregivers of under five children. Details of the youngest one were collected if there was more than one child who had had fever in a household in last 2 weeks.

### Data collection and quality assurance

The data were collected using structured interviewer administered questionnaire. Trained research assistants, with a diploma in nursing administered a questionnaire to participants individually in their homes. The questionnaire was designed in English, and translated into local languages (Amharic, Gumuz, Sinash and Agew) widely spoken in the area to facilitate the interviews. The questionnaire covered caregivers’ knowledge and practices related to malaria transmission, symptoms, treatment and preventive methods. In addition, caregivers were asked whether the child experienced an episode of fever within 2 weeks preceding the interview and related practices of treatment-seeking behaviour. The other variables considered to be associated with treatment-seeking included socio-demographic factors, perceived susceptibility, severity, barriers, and benefits as described in the HBM [22].

In order to reduce recall bias, households without fever in the last 2 weeks were just interviewed for socio-demographic characteristics and malaria knowledge but for treatment-seeking and other related factors. Two supervisors and eight data collectors participated in the data collection process. Three days training was given to the data collectors and supervisors. The supervisors checked each questionnaire and discussed with data collectors for any clarifications.

### Variables

Varying methods of calculating composite scores and categorizing them from quantitative knowledge data have increasingly been used [19]. In this study, caregiver’s malaria knowledge was measured based on a cumulative response on ten questions comprised of sign and symptoms, causes, mode of transmission, prevention and treatment of the disease. Mean score for the ten items was calculated as 7.1 (±SD 2.2) and categorized as poor and good knowledge based on the mean score below and above, respectively.

According to the HBM, the likelihood that an individual engages in a given health (enhancing) behaviour is a function of the perceived susceptibility, perceived severity, perceived treatment benefits, perceived barriers to treatment, cue to action and self-efficacy. There were six questions for perceived susceptibility, seven questions for cues to action and five questions to measure caregivers’ perceived severity of malaria. Perceived benefits of malaria treatment, perceived barriers to treatment-seeking and self-efficacy was measured using five, eight and three, respectively. The responses were ranked using 4-point Likert scale (1-strongly agree, 2-disagree, 3-agree, and 4-strongly agree). The mean scores for all constructs were computed and dichotomized into high and low. Respondent scores below the mean were labeled as having low perception of severity, susceptibility, benefits and barriers and those respondents who score mean and above categorized into high perceived severity and susceptibility of malaria disease, high perceived benefits of malaria treatment and high perceived impediments to treatment-seeking. Similarly, respondents who score below mean to questions related to cue to action and self-efficacy were categorized to have low cues to action and those who score mean and above were categorized as having high self-efficacy and high cues to action. The reliability of items of the scales were evaluated using Cronbach’s alpha. All the Cronbach’s alpha were found to be above the acceptable internal consistency level of 0.7 and above [23].

The outcome variable was caregivers’ treatment-seeking behaviour, operationally defined as seeking treatment from trained personnel or at a health facility. Caregivers were considered as seeking treatment when they visited any health facilities (Hospital, Health Center, Health Post, Private Clinic) after perceiving the illness of their child within 2 weeks of data collection period.

### Data analysis

Data were entered and cleaned using EPi-Info version 3.5.2 (Centers for Disease Control and Prevention, Atlanta, GA) and exported to SPSS version 20 program for analysis. Descriptive statistics were used to summarize all variables of interest in the study population. The statistical association between seeking treatment and each of the explanatory factors were first checked using binary logistic regression. Multivariate logistic regression was used to control confounding effects. P value ≤0.20 was taken as a cut-off point for selecting variables for the logistic regression model. Both backwards and forward logistic regression was performed and gave similar result. P values ≤0.05 were considered statistical significant in the final model. The results of all logistic regression analyses are reported as odds ratios (OR) with 95% confidence intervals (CIs). The final multivariate model was tested for goodness of fit with the Hosmer–Lemeshow test.

## Results

### Socio-demographic characteristics of study population

A total of 491 caregivers were interviewed making the response rate 97.6%. The socio-demographic characteristics of the respondents are shown in Table 1. Most of the caregivers were female (96.9%), married (96.1%) and residing in rural villages (65%). The median age was 29 years (IQR 25:32) and 61.5% were in the age group 25–34 years. In terms of educational background, 75.6% of the caregivers had no formal education, and 63.1% were not able to read and write. Most of the participants were from Gumuz (42.4%) and Agew (42.2%) ethnic origin. Over 56% of the participants were Orthodox Christians, while 24% were followers of traditional religion called ‘Gumuza Muses’. Regarding their income, about 194 (39.5%) caregivers had monthly income less than 500 Ethiopian Birr (ETB), followed by those earning 501–1000 ETB per month.

**Table 1:** Socio-demographic characteristics of caregivers of under-five children in Mandura District, Benishangul Gumuz region, West Ethiopia, December 2014

Variable name	Variable groups	All households (n = 491)	Households with fever in previous 2 weeks (n = 197)	P value
Age (IQR 25:32)	18–24	88 (17.9%)	43 (21.8%)	
	25–34	302 (61.5%)	115 (58.4%)	
	35–44	94 (19.1%)	38 (19.3%)	
	45–55	7 (1.4%)	1 (0.5%)	0.153
Sex	Male	15 (3.1%)	3 (1.5%)	0.106
	Female	476 (96.9%)	193 (98.5%)	
Residence	Urban	172 (35%)	80 (40.8%)	
	Rural	319 (65%)	117 (59.4%)	*0.034*
Educational status	Illiterate	310 (63.1%)	119 (60.4%)	
	Read and write	61 (12.4%)	28 (14.2%)	
	Primary	67 (13.6%)	28 (14.3%)	
	Secondary or above	53 (10.8%)	22 (11.2%)	0.716
Religion	Christian	323 (65.8%)	135 (68.5%)	
	Muslim	50 (10.2%)	19 (9.6%)	
	Traditional	118 (24.0%)	43 (21.8%)	0.567
Occupation	Farmer	157 (32.0%)	55 (27.9%)	
	Housewife	283 (57.6%)	121 (61.4%)	
	Merchant/employed	51 (10.4%)	21 (10.7%)	0.282
Marital status	Single	2 (0.4%)	1 (0.5%)	
	Married	472 (96.1%)	190 (96.4%)	
	Divorced	13 (2.6%)	5 (2.6%)	
	Widowed	2 (0.4%)	0	
	Separated	2 (0.4%)	1 (0.5%)	
Family size	<6	289 (60.4%)	119 (60.4%)	
	≥6	202 (25.7%)	78 (39.6%)	0.569
Ethnicity	Gumuz	208 (42.4%)	80 (40.8%)	
	Agew	207 (42.2%)	86 (43.9%)	
	Shinasha	7 (1.4%)	0	
	Amhara	65 (13.2%)	30 (15.3%)	
	Tigre	1 (0.2%)	0	
	Oromo	3 (0.6%)	0	
Average monthly income (in ETB)	<500 (~25 USD)	194 (39.5%)	78 (39.6%)	
	500–1000 (~25–50 USD)	120 (24.4%)	46 (23.4%)	
	>1000 (~>50 USD)	177 (36.0%)	73 (37.0%)	0.881

*ETB* Ethiopian birr, *USD* US dollars

Socio-demographic characteristics were similar between households visited and households with febrile under five children except for educational status (Italic)

### Knowledge of caregivers about malaria

A series of questions regarding risk factors, symptoms, prevention, early detection and treatment options were asked to determine caregivers’ level of knowledge about malaria. Majority (70.9%) of the participants associated malaria with mosquito bite. However, some associated malaria transmission with cold wet weather (21.8%) and consumption of contaminated food and drinking (1.2%). Mosquito breeding site was reported by majority (84.5%) as stagnant water and 4.9% reported running water as common breeding site.

Most respondents were aware of the common signs and symptoms of malaria in both adults and children. The most common frequently perceived signs and symptoms of malaria included hot body/fever (94.1%), followed by headache (82%). The vast majority (96.7) of the study participants knew that malaria is preventable. Using bed net (91%), eliminating mosquito breeding sites (57.2%), DDT spray (51.5%) and using personal protection (35.2%) were the most frequently mentioned prevention methods. Using the sum of all knowledge items, 45% of the participants were found to have sufficient knowledge about malaria. About 88.4% of caregivers reported modern health institution as the best option to treat malaria in under five children, while traditional medicine was mentioned by 11.4% of the participants.

### Caregivers perception on malaria

Overall, 56.2% of caregivers had low perception about susceptibility to malaria infection and 51.1% had low perception about severity of the disease. Nearly 63.7% of caregivers had low perceived barrier to seek treatment for under-five children. However, 51.3% of caregivers had low perception of the benefits of seeking treatment. More than 60% of respondents had low cue to action and 58.7% had low self-efficacy. Details about the constructs of the HBM are shown in Table 2.

**Table 2:** Descriptive statistics for HBM constructs

Variable name	Variable groups	Strongly agree	Agree	Disagree	Strongly disagree
Perceived severity (Cronbach’s α = 0.79)	Malaria is a serious disease in children	277 (56.4)	196 (39.9)	8 (1.6)	10 (2.0)
	Worried that my child was suffering from malaria	10 (2.0)	69 (14.1)	171 (34.8)	241 (49.1)
	Complications of malaria are dangerous and result in death	241 (49.1)	227 (46.2)	14 (2.9)	9 (1.8)
	Risk of death from malaria is higher in children compared to adults	235 (47.9)	234 (47.7)	14 (2.9)	8 (1.6)
	Malaria treatment costs me more money if complicated	211 (43.0)	257 (52.3)	15 (3.1)	8 (1.6)
Perceived susceptibility (Cronbach’s α = 0.89)	Persistent vomiting and diarrhea could be due to malaria	195 (39.7)	249 (50.7)	39 (7.9)	8 (1.6)
	Children would be unable to eat or have poor appetite due to malaria	184 (37.5)	292 (59.5)	9 (1.8)	6 (1.2)
	Fever could be due to malaria	214 (43.6)	264 (53.8)	7 (1.4)	6 (1.2)
	Malaria can cause anemia	141 (28.7)	282 (57.4)	58 (11.8)	10 (10.2)
	Malaria could cause convulsion, chilling and joint pain	190 (38.7)	275 (56.0)	20 (4.1)	6 (1.2)
	Children always have a chance to be infected with malaria	167 (34.1)	258 (52.5)	61 (12.4)	5 (1.0)
Perceived benefits (Cronbach’s α = 0.87)	Child will get better as soon as if taken to health facility	246 (50.1)	224 (45.6)	17 (3.5)	4 (0.8)
	Taking a child to a health facility prevents further complications	177 (36.0)	295 (60.1)	16 (3.3)	3 (0.6)
	Seeking treatment avoids additional cost to treat complications	185 (37.7)	292 (59.5)	9 (1.8)	5 (1.0)
	Seeking treatment reduce the chance of death	170 (34.6)	309 (62.9)	8 (1.6)	4 (0.8)
	Value spending money spent for child treatment seeking	152 (31.0)	302 (61.5)	33 (6.7)	4 (0.8)
Perceive barrier (Cronbach’s α = 0.82)	The drugs are not effective to treat malaria	2 (0.4)	8 (1.6)	35 (7.1)	446 (90.8)
	Malaria subsides by itself without treatment	1 (0.2)	13 (2.6)	56 (11.4)	421 (85.7)
	Health facility is far from where we live	1 (0.2)	9 (1.8)	48 (9.8)	433 (88.2)
	Have no money to take the child to health facility	8 (1.6)	64 (13.0)	112 (22.8)	307 (62.5)
	Traditional healers can treat the child with fever/malaria	2 (0.4)	13 (2.6)	49 (10.0)	427 (87.0)
	The disease is not serious enough	1 (0.2)	13 (2.6)	32 (6.5)	445 (90.6)
	Home treatment is sufficient	4 (0.8)	13 (2.6)	44 (9.0)	430 (87.6)
	Long waiting time at health facility	2 (0.4)	11 (2.2)	53 (10.8)	425 (86.6)
Cue to action (Cronbach’s α = 0.89)	Malaria related messages broadcasted on television	107 (21.8)	204 (41.5)	142 (28.9)	38 (7.7)
	Malaria related messages broadcasted on radio	105 (21.4)	273 (55.6)	86 (17.5)	27 (5.5)
	Advise from health workers	209 (42.6)	272 (55.4)	6 (1.2)	4 (0.8)
	Advise from peers	148 (30.1)	304 (61.9)	27 (5.5)	12 (2.4)
	Advise from health extension workers	151 (30.8)	291 (59.3)	38 (7.7)	11 (2.2)
	Advise from family members	146 (29.7)	290 (59.1)	46 (9.4)	9 (1.8)
	History of death of a child from malaria	134 (27.3)	262 (53.4)	61 (12.4)	34 (6.9)
Self-efficacy (Cronbach’s α = 0.92)	Distinguish malaria from other illnesses	208 (42.4)	270 (55.0)	8 (1.6)	5 (1.0)
	Can consult health workers for fever/malaria	195 (39.7)	287 (58.5)	8 (1.6)	1 (0.2)
	Can use ITN to reduce risk of malaria infection in children	193 (39.3)	285 (58.0)	12 (2.4)	1 (0.2)

### Treatment-seeking behaviours

From the total number of children under-five (197) with fever in the past 2 weeks, 173 (87.8%) were brought to treatment. Of those children brought to treatment, 129 (74.6%) received treatment in the public health institution and 44 (25.4%) received treatment from local private providers like drug vendors. Some of the children (18.5%) received treatment at home before they were taken to health facility. Majority (61.3%) of the children sought treatment at least 24 h after the onset fever. The mean hours the child brought to the health institution was 5 days after the onset of fever. Moreover, some of the caretakers (18.5%) first consulted traditional healers and used home remedies for malaria treatment in their children.

### Factors affecting treatment-seeking behaviour

In the bivariate analysis, among the socio-demographic characteristics of respondents, age, residence, ethnicity, occupation, and religion were significantly associated with malaria treatment-seeking behaviour. Of the malaria perception variables, perceived susceptibility for malaria, perceived barriers to treatment-seeking and cues to action were found to be significantly associated with malaria treatment-seeking behaviour.

The results of multivariate logistic regression analysis showed that five explanatory variables were independently associated with malaria treatment-seeking for under-five children suspected of having malaria (Table 3). Age of caregivers appears to be a significant determinant for seeking treatment. Younger caregivers (age <30 years) were 3.40 more likely (95% CI 1.27–9.10) to seek treatment as compared to those >30 years of age, adjusted for other variables. As compared with urban residents, caregivers from rural area were more likely to seek treatment for under five children, adjusting for other variables (AOR 2.80, 95% CI 1.01, 7.70).

**Table 3:** Factors associated with caregivers’ treatment-seeking behaviour for under five children with fever (n = 197)

Variable	Variable groups	Treatment sought (%)	COR (95% CI)	AOR (95% CI)
Age of caregivers	<30	103 (92.0)	2.5 (1.02–5.91)*	3.40 (1.27–9.10)**
	>30	70 (82.4)	1.00	1.00
Place of residence	Rural	107 (91.5)	2.27 (0.95–5.40)*	2.80 (1.01–7.70)**
	Urban	66 (82.5)	1.00	1.00
Educational status	Illiterate	103 (86.6)	1.00	
	Read and write	26 (92.9)	2.02 (0.44–9.34)	
	Primary	25 (89.3)	1.29 (0.26–4.79)	
	Secondary or above	19 (86.4)	0.98 (0.26–3.71)	
Religion	Christian	121 (89.6)	1.00	
	Muslim	18 (94.7)	2.08 (0.26–16.81)	
	Traditional	34 (79.1)	0.44 (0.17–1.10)*	
Ethnicity	Gumuz	66 (82.5)	1.00	
	Agew	79 (90.8)	2.09 (0.83–5.30)*	
	Amhara	28 (93.3)	2.97 (0.63–13.94)*	
Family size	≤5	106 (89.1)	1.34 (0.57–3.16)	
	≥6	67 (85.9)	1.00	
Occupation of respondents	Farmer	45 (81.8)	1.00	
	Housewife	109 (90.5)	2.02 (0.81–5.01)*	
	Merchant/government employee	19 (90.5)	2.11 (0.42–10.56)	
Average monthly income (ETB)	≤500	68 (87.2)	1.00	
	501–1000	42 (91.3)	1.54 (0.46–5.24)	
	>1000	63 (86.3)	0.93 (0.36–2.37)	
Malaria knowledge	Poor	95 (82.6)	1.00	1.00
	Good	78 (95.1)	4.11 (1.35–12.51)*	4.65 (1.38–15.64)**
Perceived susceptibility	Low	99 (84.6)	1.00	1.00
	High	74 (92.5)	2.24 (0.85–5.93)*	3.63 (1.21–10.88)**
Perceived severity	Low	87 (86.1)	1.00	
	High	84 (87.5)	0.94 (0.40–2.22)	
Perceived benefits of malaria treatment	Low	87 (86.1)	1.00	
	High	86 (89.6)	1.38 (0.58–3.29)	
Perceived barriers to treatment-seeking	Low	106 (93.0)	1.00	1.00
	High	67 (80.7)	0.32 (0.13–0.78)*	0.18 (0.06–0.52)**
Cues to action	Low	102 (85.0)	1.00	
	High	71 (92.2)	2.09 (0.79–5.52)*	
Perceived self-efficacy	Low	106 (87.6)	1.00	
	High	67 (88.2)	1.05 (0.44–2.54)	

*COR* crude odds ratio, *AOR* adjusted odds ratio, *CI* confidence interval

* P < 0.2, ** P < 0.05; No odds ratio was calculated for sex of respondents since all of the male respondents sought treatment

Caregivers’ malaria knowledge was also a significant predictor of treatment-seeking behaviour. Children whose caregivers had good malaria knowledge were more likely to be taken for medical care (AOR 4.65, 95% CI 1.38–15.64). Caregivers who had high perception that their child could be susceptible to malaria were more likely to seek treatment (AOR 3.63, 95% CI 1.21–10.88). Moreover, caregivers who had high perceived barriers to seek treatment were less likely to bring their children for medical attention (AOR 0.18, 95% CI 0.06–0.52).

## Discussion

This study assessed caregivers’ perception about malaria in view to determine treatment-seeking behaviour for their children under five with fever and associated factors. The results indicate that almost all the study participants knew malaria and associated it with mosquito bite. Majority of the participants were aware of the common signs and symptoms of malaria and reported that malaria is preventable. However, only 45% of the participants had sufficient knowledge about malaria and significant number of respondents had some misconception regarding causes of the disease. The study found out that 87.8% of the children under 5 years of age with fever were taken to formal health care facility.

The majority of the caregivers reported that malaria could be transmitted from person-to-person through mosquito bites, which is comparable to another study in Ethiopia [24]. This finding is however higher when compared to a recent study conducted in Nigeria where 35.2% correctly identified mosquito bite as the main cause of malaria [25]. Majority of the respondents have also mentioned fever as the most frequently recognized symptom of childhood malaria, this finding corroborates with other study in Ethiopia [24]. Likewise, majority of the participants were aware of the most common preventive methods of malaria and mentioned modern anti-malarial drugs as the best treatment for the disease. However, only 45% of the participants had sufficient knowledge about malaria. This finding indicates the need to enhance caregivers’ malaria knowledge as it has also been reported to influence treatment-seeking behaviour [26].

On the other hand, a considerable number of the caregivers had misconceptions on causation of malaria. In some cases, malaria was associated to exposure to cold wet weather and consumption of contaminated food/water. Misconceptions about the spread of the disease have also been documented in several studies in malarias areas of Ethiopia [24] and other African settings [27]. Such belief would have a negative influence on prompt care seeking and treatment sources preferences. It is possible that these misconceptions affect caregivers’ initial decision to seek medical care and hamper prevention and control of the disease.

A large proportion of the children with fever were brought to treatment, which is consistent with a study in Myanmar [26]. While early detection and access to prompt treatment is regarded as the corner stone of a successful malaria control strategy, however, most children sought treatment after 24 h of onset of fever, thus, in the late stage of the disease. Moreover, a considerable number of caregivers first consulted traditional healers and continued up to the health institution when complete cure was not yet achieved. Delays of more than 24 h in care seeking has also been reported in other African settings [28–30]. Thus, more could be done to ensure prompt malaria treatment to prevent death since the majority of malaria deaths occur within the first 24 h following onset of fever [31], which is also emphasized in the Ethiopian malaria prevention and control strategy [32]. Previous studies have recommended involvement of traditional healers in malaria treatment programs and encouraged them to incorporate the use of evidence-based anti-malarials into their practice [33].

Contrary to researches reported elsewhere [19, 34, 35], in this study, caregivers who were residing in rural areas were more likely to seek health care than their urban counterparts. The current finding is consistent with a report from Uganda in which children in rural areas were found to seek health care more than their urban counterparts [36]. This finding may be explained by the fact that urban dwellers are relatively more educated, with better access to pharmacies and drug shops and thus the tendency to self-treat. Studies have reported that perceived efficacy is related with tendency for self-treatment of malaria [37]. The finding could also show improved treatment-seeking behaviour in rural settings attributed to implementation of integrated community case management (ICCM) through health extension workers (HEWs) in the country [38]. HEWs provide promotional and preventive services, including diagnosis and treatment of malaria among children under five, in addition to their other responsibilities [39].

The finding that knowledge of malaria is associated with treatment-seeking is corroborated by research reported elsewhere [36, 40]. Some other studies highlighted that knowledge about malaria symptoms may not be enough to explain prompt and adequate care seeking alone [17, 19, 41, 42]. The current finding is consistent with a previous study that reported malaria knowledge could improve malaria health related behaviours independent of other factors [19]. This finding could be attributed to the fact that early treatment depends upon prompt recognition of symptoms and signs of malaria in the household mainly by women [10, 16]. It appears that caregiver’s knowledge about malaria treatment is essential to improve health seeking behaviours.

Children whose caregivers were below 30 years of age were more likely to receive treatment than children whose mothers were older. This finding is in line with reports from other African settings [43]. However, some studies have reported that older caregivers are more likely to seek appropriate health care compared to younger women [44, 45]. It is possible that older caregivers may be less likely to seek prompt and effective treatment if they are caring for multiple children. If they draw upon prior experience with febrile children, they also may be less inclined to follow new health messaging, thereby inhibiting treatment-seeking [46].

In addition, caregivers who perceived that their child could be susceptible to febrile illness were more likely to seek treatment as compared with caregivers who had low perception about susceptibility. This finding is in line with the report of a study in Oromia region of Ethiopia [47]. Similarly, significant relationship was found between perceived barrier and malaria treatment-seeking behaviour. Caregivers who had high perceived barriers were less likely to have higher odds of treatment-seeking for under five children. Whereas in a recent study in Ethiopia perceived barriers was not associated with treatment-seeking behaviour [47]. The discrepancy could be due to the difference in socio-cultural difference of the respondents.

This study has some limitations that should be considered. First, this study was unable to explore causal relationships because of a cross-sectional study design. Secondly, the study team interviewed caregivers to obtain data on presumed fever/malaria cases at the household level. However, earlier studies have shown that caretakers generally have a good understanding of febrile illnesses in terms of types and symptoms. Lastly, data presented is based on participants’ self-reports, which may be associated with socially desirable bias.

## Conclusions

In conclusion, the majority of respondents have knowledge on sign and symptom of malaria as well as its prevention and control methods but some caregivers of children under-five had misconceptions about the cause and transmission of the disease. Majority of the respondents of this study sought treatment for their under-five children. However, a considerable number of caretakers first consulted traditional healers and tried home treatment, thus, sought treatment late. The study revealed several determinants of malaria treatment-seeking for under-five children including caregivers’ age, malaria knowledge, perceived severity of malaria and perceived barrier to seek treatment. There is a need to focus on targeted interventions, promote awareness and prevention, and address misconceptions about childhood febrile illness.
